# Supplementary figures and images for: Elucidating the mechanism by which synthetic helper peptides sensitize Pseudomonas aeruginosa to multiple antibiotics
Source: PLoS Pathog. 2021 Sep 3;17(9):e1009909. doi: 10.1371/journal.ppat.1009909 (PMC8445441; doi:10.1371/journal.ppat.1009909)

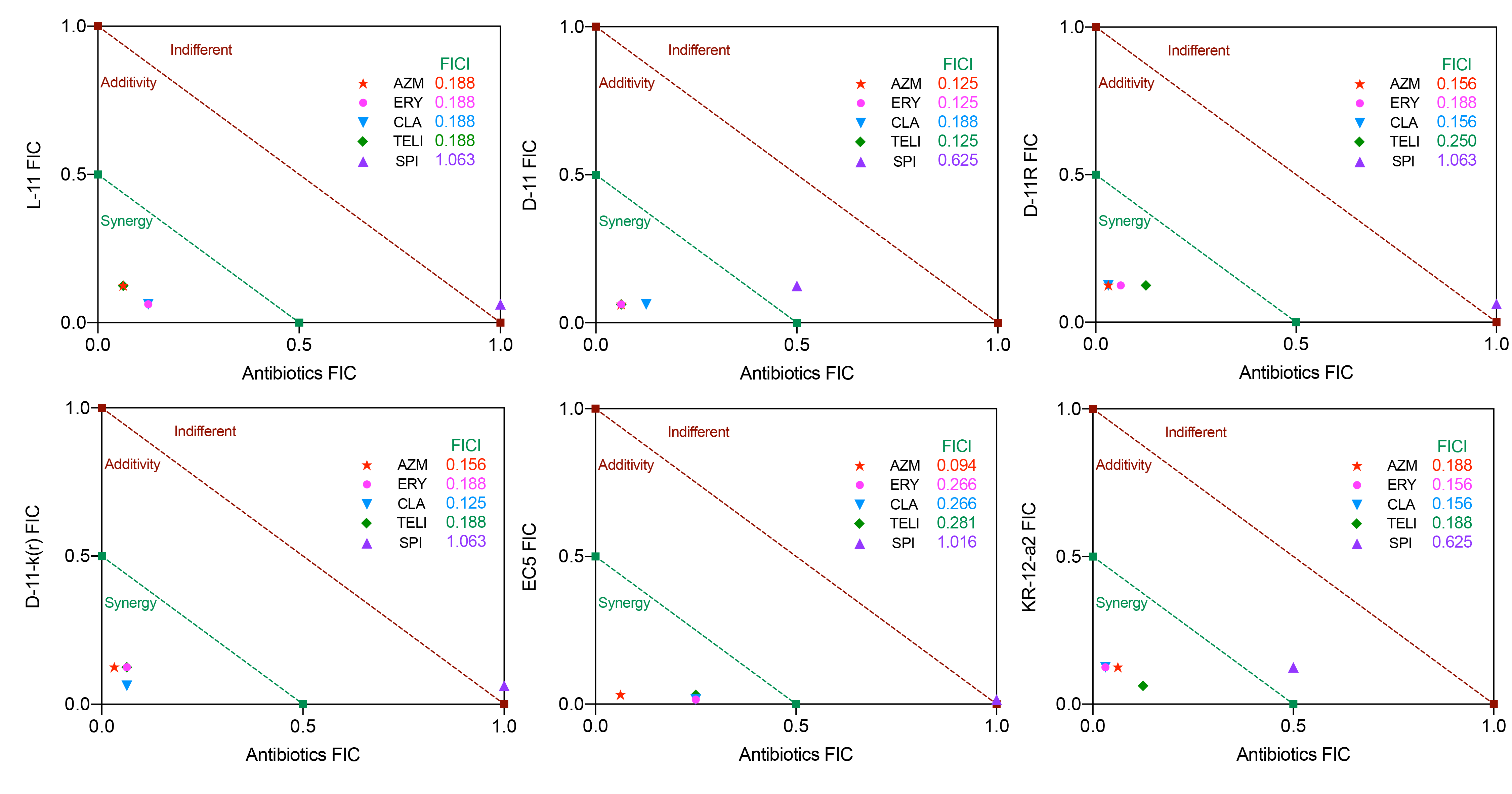

Supplement: S1 Fig — The FICI plot for synergistic peptides and macrolides against PAO1. AZM, azithromycin; ERY, erythromycin; CLA, clarithromycin; TELI, telithromycin; SPI, spiramycin. (TIF) [file ppat.1009909.s001.tif]

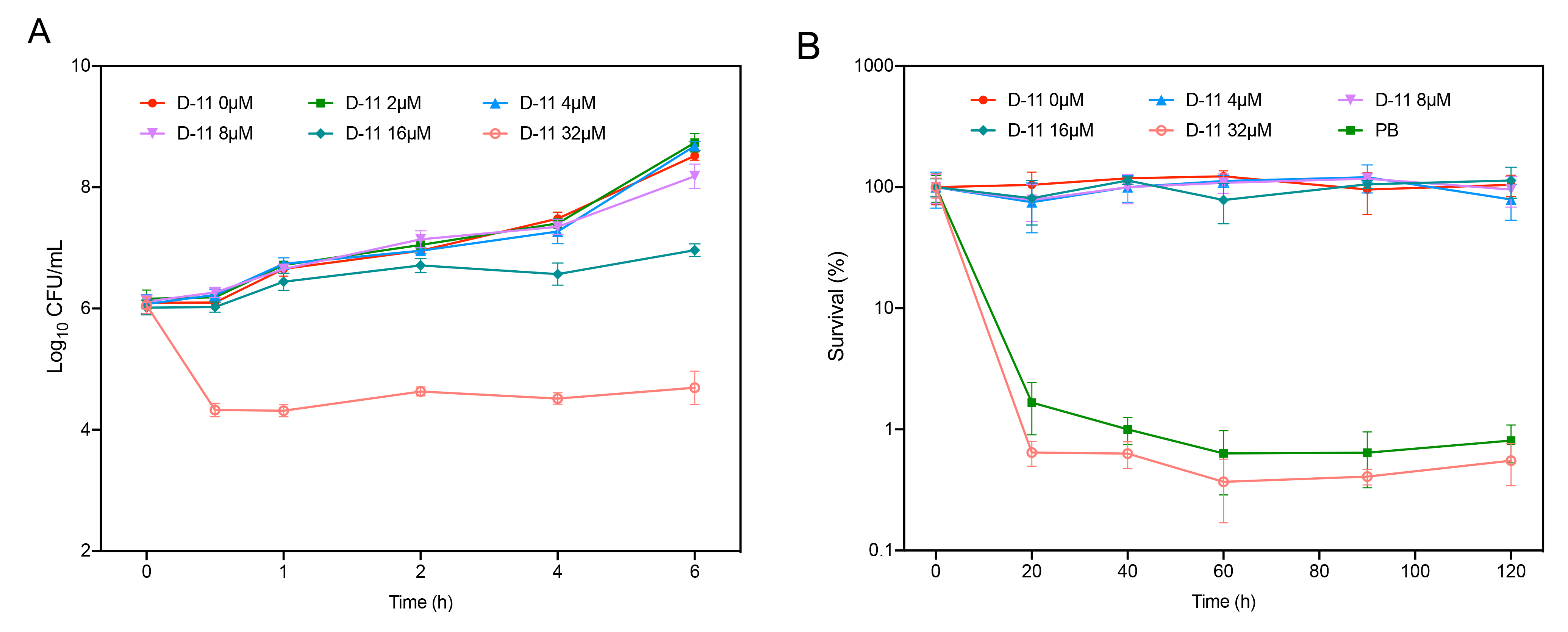

Supplement: S2 Fig — (A) Time killing curves of D-11 against PAO1 during 6 h incubation at the indicated concentration. (B) Survival rates of bacteria under different treatment conditions during PI uptake assay. (TIF) [file ppat.1009909.s002.tif]

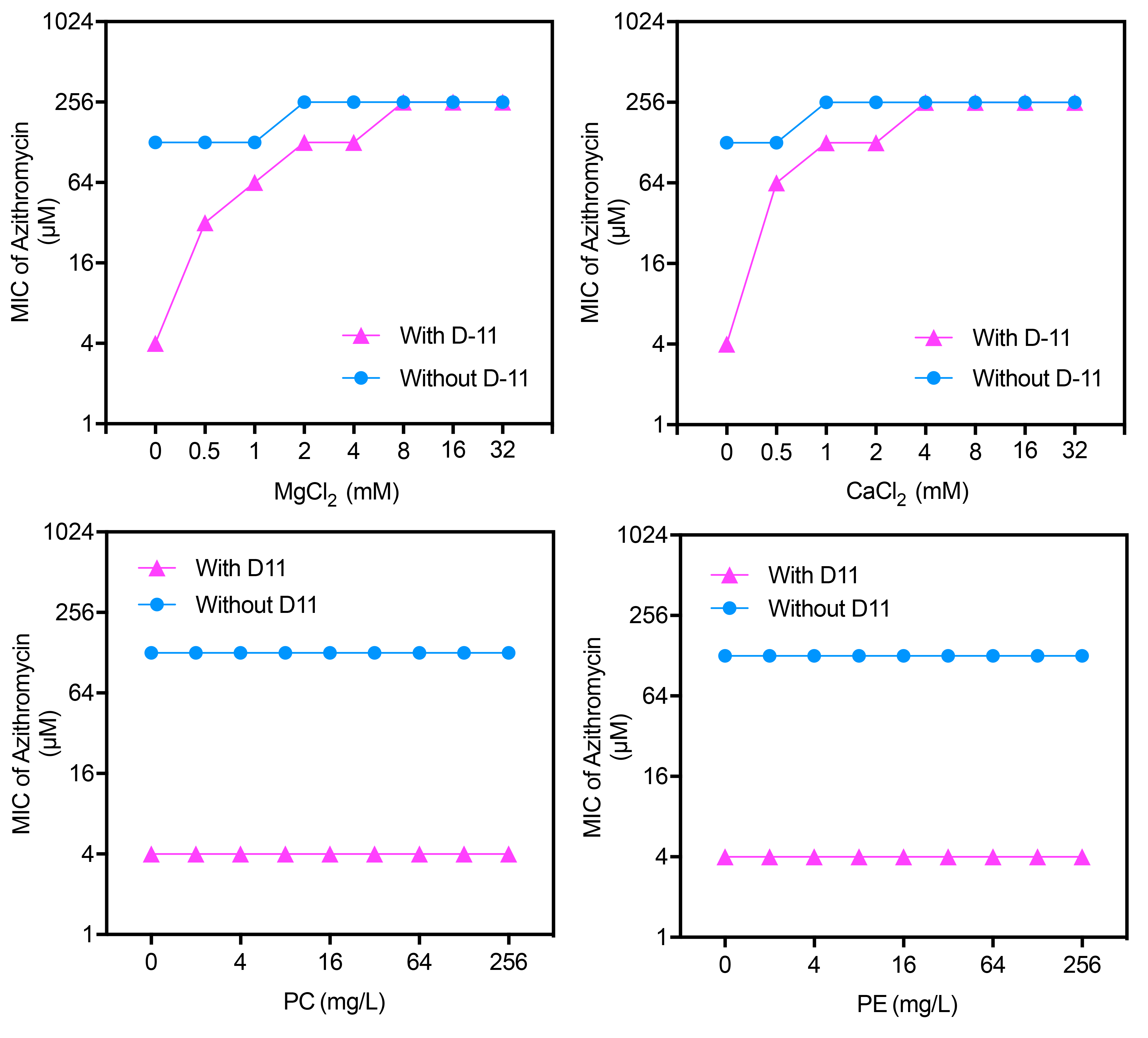

Supplement: S3 Fig — Effect of cationic divalent as MgCl2 (A) and CaCl2 (B) on the synergistic activity of D-11 and azithromycin. The MIC of azithromycin was determined by checkerboard microdilution assays in the presence of MgCl2 or CaCl2 (0–32 mM) with or without 4 μM D-11. Effect of PC (C) and PE (D) on D-11 and azithromycin combination. (TIF) [file ppat.1009909.s003.tif]

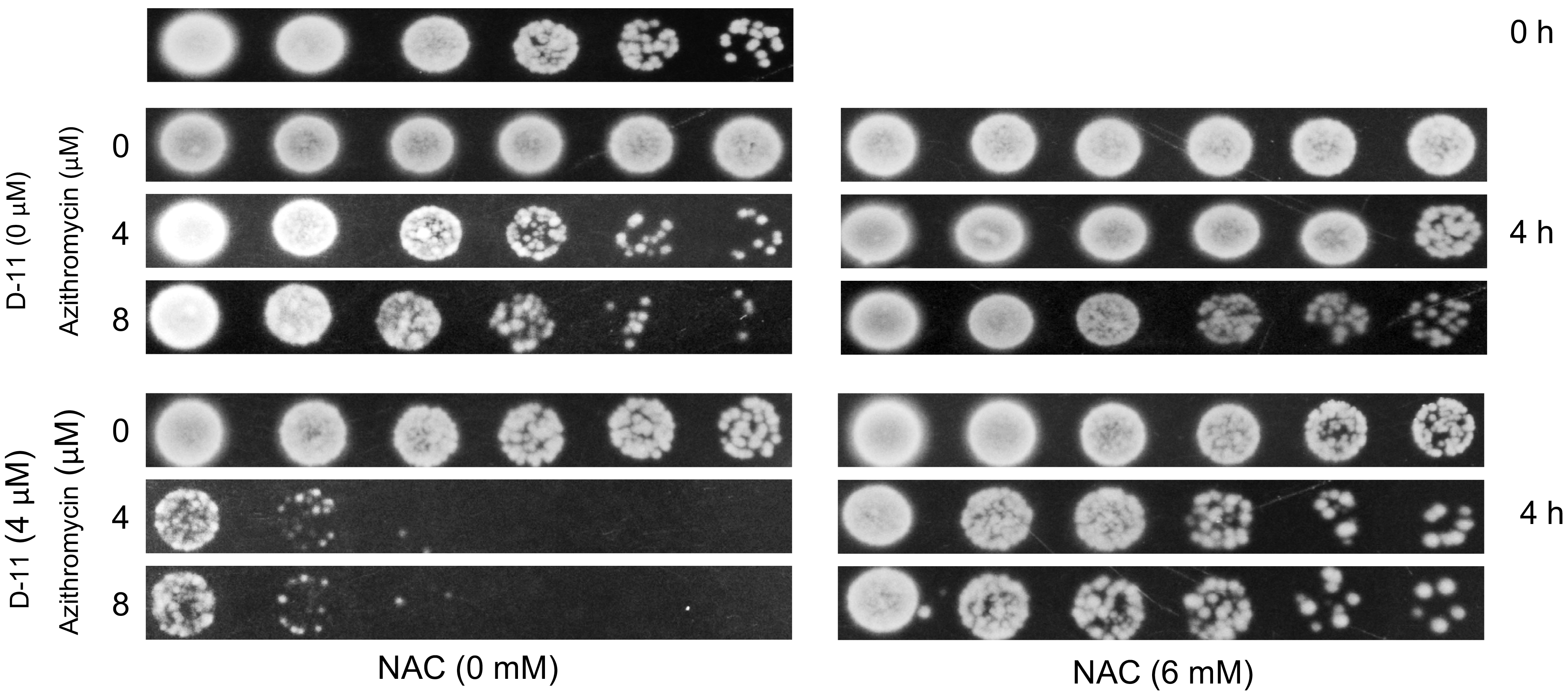

Supplement: S4 Fig — (TIF) [file ppat.1009909.s004.tif]

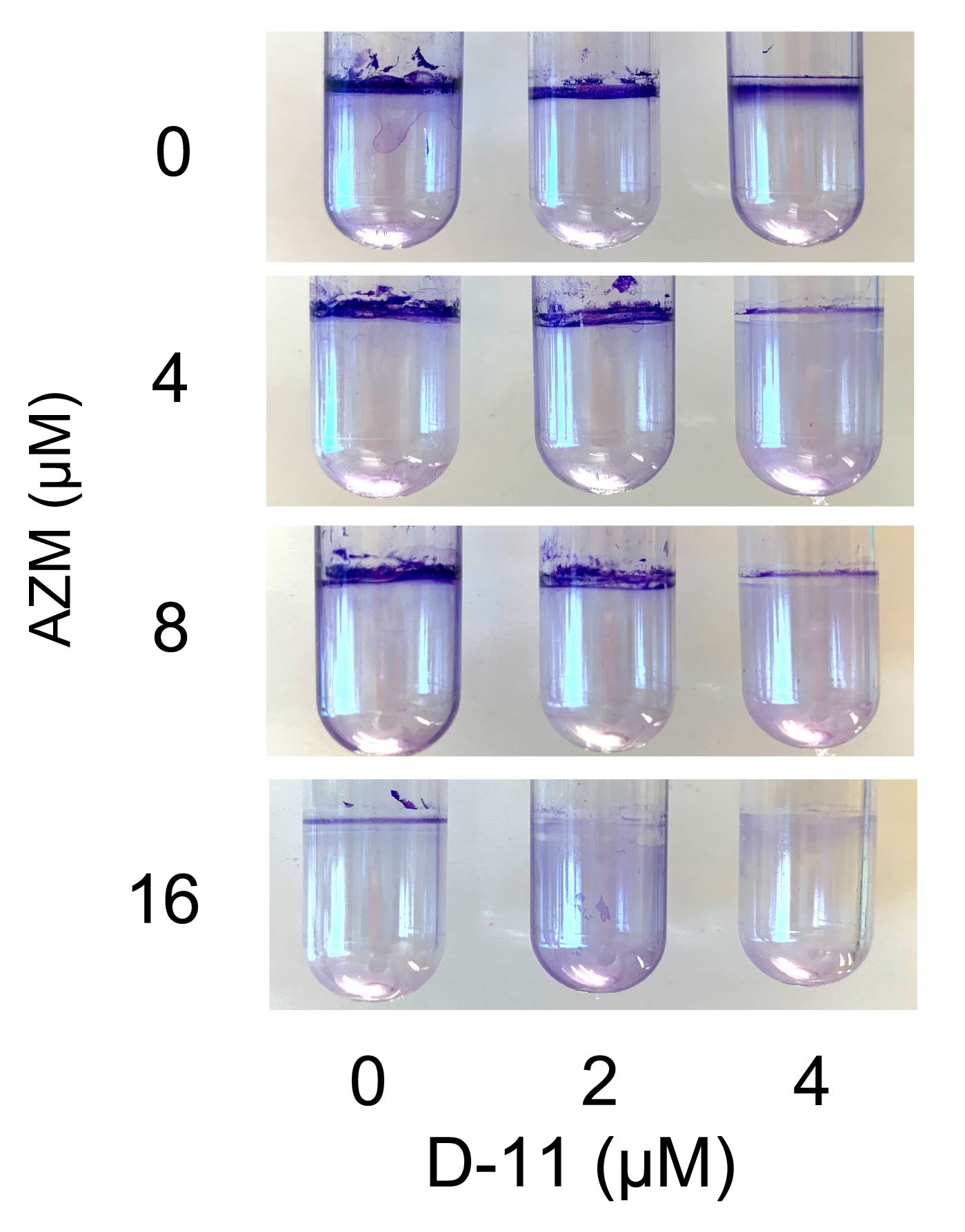

Supplement: S5 Fig — (TIF) [file ppat.1009909.s005.tif]

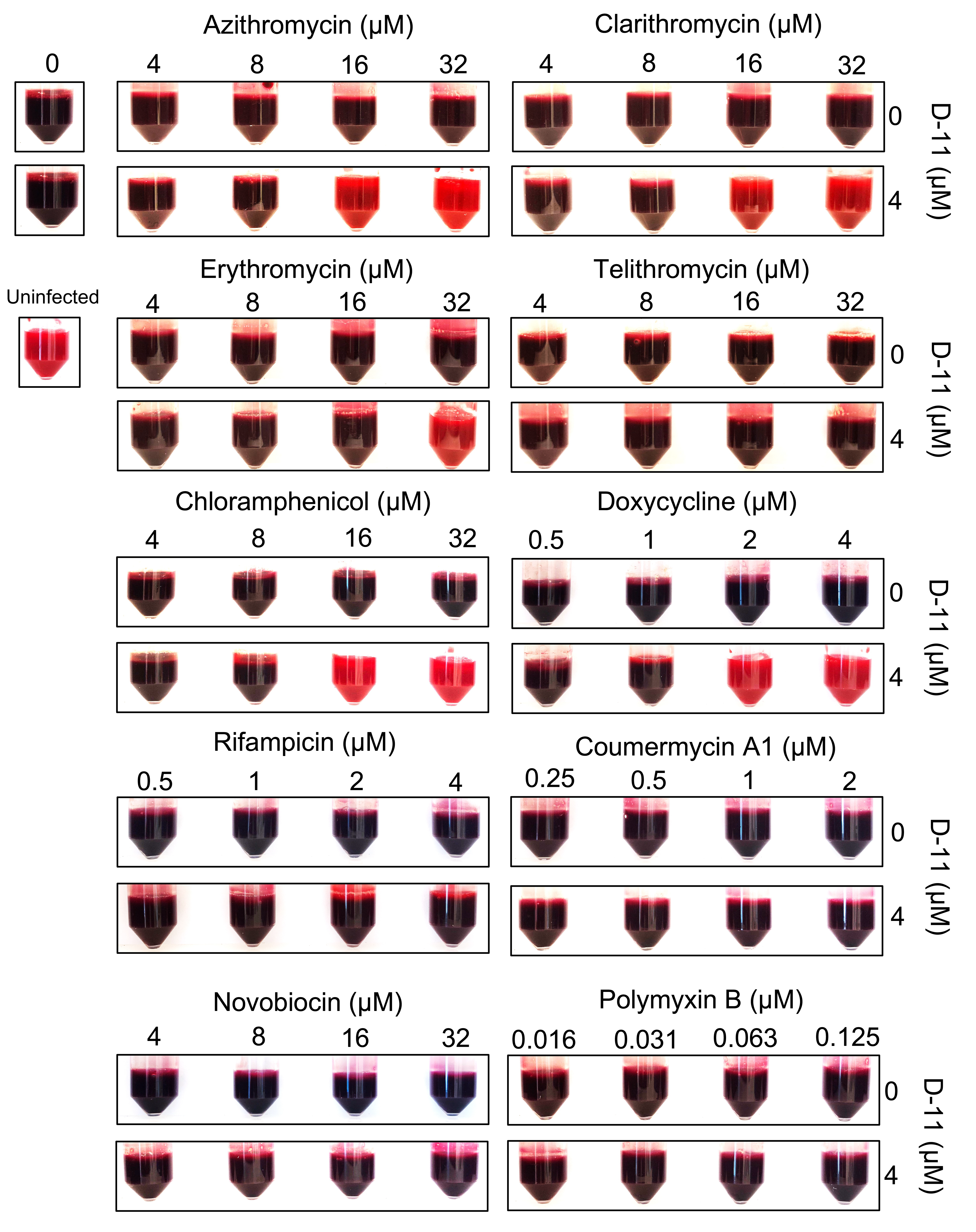

Supplement: S7 Fig — Black colour of the blood indicates hemolysis and therefore bacterial growth. (TIF) [file ppat.1009909.s007.tif]
