# Supplementary material for: Elucidating the mechanism by which synthetic helper peptides sensitize Pseudomonas aeruginosa to multiple antibiotics
Source: PLoS Pathog. 2021 Sep 3;17(9):e1009909. doi: 10.1371/journal.ppat.1009909 (PMC8445441; doi:10.1371/journal.ppat.1009909)
Supplement: S1 Table — (DOCX) [file ppat.1009909.s008.docx]

**S1 Table. The synergy effects of peptides and macrolides against PAO1.**

| Peptides | Antibiotics | MICa/MICac (μM) | MICb/MICbc (μM) | FICI |  |
| --- | --- | --- | --- | --- | --- |
|  |  |  |  |  |  |
| L-11 | Azithromycin | 128/16 | 32/2 | 0.188 |  |
|  |  | 128/8 | 32/4 | 0.188 |  |
|  | Clarithromycin | 128/16 | 32/2 | 0.188 |  |
|  |  | 128/8 | 32/4 | 0.188 |  |
|  | Erythromycin | 128/16 | 32/2 | 0.188 |  |
|  |  | 128/8 | 32/4 | 0.188 |  |
|  | Spiramycin | >128/>128 | 32/2 | 1.063 |  |
|  |  | >128/>128 | 32/4 | 1.125 |  |
|  | Telithromycin | 64/8 | 32/2 | 0.188 |  |
|  |  | 64/4 | 32/4 | 0.188 |  |
| D-11 | Azithromycin | 128/8 | 32/2 | 0.125 |  |
|  |  | 128/4 | 32/4 | 0.156 |  |
|  | Clarithromycin | 128/16 | 32/2 | 0.188 |  |
|  |  | 128/8 | 32/4 | 0.188 |  |
|  | Erythromycin | 128/8 | 32/2 | 0.125 |  |
|  |  | 128/2 | 32/4 | 0.141 |  |
|  | Spiramycin | >128/>128 | 32/2 | 1.063 |  |
|  |  | >128/128 | 32/4 | 0.625 |  |
|  | Telithromycin | 64/4 | 32/2 | 0.125 |  |
|  |  | 64/2 | 32/4 | 0.156 |  |
| D-11R | Azithromycin | 128/16 | 32/2 | 0.188 |  |
|  |  | 128/4 | 32/4 | 0.156 |  |
|  | Clarithromycin | 128/32 | 32/2 | 0.313 |  |
|  |  | 128/4 | 32/4 | 0.156 |  |
|  | Erythromycin | 128/16 | 32/2 | 0.188 |  |
|  |  | 128/8 | 32/4 | 0.188 |  |
|  | Spiramycin | >128/>128 | 32/2 | 1.063 |  |
|  |  | >128/>128 | 32/4 | 1.125 |  |
|  | Telithromycin | 64/16 | 32/2 | 0.313 |  |
|  |  | 64/8 | 32/4 | 0.250 |  |
| D-11-k78r | Azithromycin | 128/16 | 64/4 | 0.188 |  |
|  |  | 128/4 | 64/8 | 0.156 |  |
|  | Clarithromycin | 128/8 | 64/4 | 0.125 |  |
|  |  | 128/4 | 64/8 | 0.156 |  |
|  | Erythromycin | 128/16 | 64/4 | 0.188 |  |
|  |  | 128/8 | 64/8 | 0.188 |  |
|  | Spiramycin | >128/>128 | 64/4 | 1.063 |  |
|  |  | >128/>128 | 64/8 | 1.125 |  |
|  | Telithromycin | 64/8 | 64/4 | 0.188 |  |
|  |  | 64/4 | 64/8 | 0.188 |  |
| L-EC5 | Azithromycin | 128/32 | >128/4 | <0.281 |  |
|  |  | 128/8 | >128/8 | <0.125 |  |
|  | Clarithromycin | 128/32 | >128/4 | <0.281 |  |
|  |  | 128/32 | >128/8 | <0.313 |  |
|  | Erythromycin | 128/32 | >128/4 | <0.281 |  |
|  |  | 128/32 | >128/8 | <0.313 |  |
|  | Spiramycin | >128/>128 | >128/4 | 1.031 |  |
|  |  | >128/>128 | >128/8 | 1.063 |  |
|  | Telithromycin | 64/32 | >128/4 | <0.531 |  |
|  |  | 64/16 | >128/8 | <0.313 |  |
| KR-12-a2 | Azithromycin | 128/16 | 32/2 | 0.188 |  |
|  |  | 128/8 | 32/4 | 0.188 |  |
|  | Clarithromycin | 128/16 | 32/2 | 0.188 |  |
|  |  | 128/4 | 32/4 | 0.156 |  |
|  | Erythromycin | 128/16 | 32/2 | 0.188 |  |
|  |  | 128/4 | 32/4 | 0.156 |  |
|  | Spiramycin | >128/>128 | 32/2 | 1.063 |  |
|  |  | >128/128 | 32/4 | 0.625 |  |
|  | Telithromycin | 64 | 32/2 | 0.188 |  |
|  |  | 64 | 32/4 | 0.188 |  |

MICa: the MIC of antibiotics, MICb: the MIC of the peptide, MICac: the MIC of antibiotic in the combination, MICbc: the MIC of the peptide in the combination, the synergy effects were bolded in FICI. Red marks mean synergistic effects. If the MIC of peptides is higher than 128 μM, we used 256 for the calculation.
