# Supplementary material for: Elucidating the mechanism by which synthetic helper peptides sensitize Pseudomonas aeruginosa to multiple antibiotics
Source: PLoS Pathog. 2021 Sep 3;17(9):e1009909. doi: 10.1371/journal.ppat.1009909 (PMC8445441; doi:10.1371/journal.ppat.1009909)
Supplement: S3 Table — (DOCX) [file ppat.1009909.s010.docx]

**S3 Table. The screening of synergy effects of D-11 with multiple antibiotics**

| Antibiotics | MICa/MICac  (μM) | MICb/MICbc  (μM) | FICI |
| --- | --- | --- | --- |
| Amikacin | 1/1 | 32/2 | 1.063 |
|  | 1/2 | 32/4 | 2.125 |
| Amoxicin | >128/>128 | 32/2 | 1.125 |
|  | >128/>128 | 32/4 | 1.125 |
| Ampicillin | >128/>128 | 32/2 | 1.125 |
|  | >128/>128 | 32/4 | 1.125 |
| Aztreonam | 8/1 | 32/2 | 0.188 |
|  | 8/0.25 | 32/4 | 0.156 |
| Bacitracin | >128/>128 | 32/2 | 1.063 |
|  | >128/>128 | 32/4 | 1.125 |
| Carbenicillin | 64/16 | 32/2 | 0.313 |
|  | 64/4 | 32/4 | 0.188 |
| Cefepime | 2/1 | 32/2 | 0.563 |
|  | 2/1 | 32/4 | 0.625 |
| Ceftaroline | 128/128 | 32/2 | 1.063 |
|  | 128/128 | 32/4 | 1.125 |
| Cephalexin | >128/>128 | 32/2 | 1.063 |
|  | >128/>128 | 32/4 | 1.125 |
| Chloramphenicol | 128/4 | 32/2 | 0.094 |
|  | 128/4 | 32/4 | 0.156 |
| Chlorobiocin | 0.5/0.125 | 32/2 | 0.313 |
|  | 0.5/0.125 | 32/4 | 0.375 |
| Ciprofloxacin | 4/0.5 | 32/2 | 0.188 |
|  | 4/0.25 | 32/4 | 0.188 |
| Colistin | 0.25/0.004 | 32/2 | 0.079 |
|  | 0.25/0.001 | 32/4 | 0.129 |
| Coumermycin A1 | 8/0.125 | 32/2 | 0.078 |
|  | 8/0.015625 | 32/4 | 0.064 |
| Doxycycline | 16/0.5 | 32/2 | 0.094 |
|  | 16/0.25 | 32/4 | 0.141 |
| Eravacycline | 4/0.125 | 32/2 | 0.094 |
|  | 4/0.0625 | 32/4 | 0.078 |
| Fosfomycin | >128 | 32/2 | 0.188 |
|  | >128 | 32/4 | 0.188 |
| Fusaric Acid | >128/>128 | 32/2 | 1.063 |
|  | >128/>128 | 32/4 | 1.125 |
| Gentamicin | 1/2 | 32/2 | 2.063 |
|  | 1/2 | 32/4 | 2.125 |
| Kanamycin | 64/128 | 32/2 | 2.063 |
|  | 64/128 | 32/4 | 2.125 |
| Levofloxacin | 1/0.0625 | 32/2 | 0.125 |
|  | 1/0.03125 | 32/4 | 0.156 |
| Linezolid | >128/>128 | 32/2 | 1.063 |
|  | >128/>128 | 32/4 | 1.125 |
| Loperamide | >128/>128 | 32/2 | 1.063 |
|  | >128/>128 | 32/4 | 1.125 |
| Meropenem | 2/1 | 32/2 | 0.625 |
|  | 2/0.5 | 32/4 | 0.375 |
| Metronidazole | >128/>128 | 32/2 | 1.063 |
|  | >128/>128 | 32/4 | 1.125 |
| Minocycline | 16/0.5 | 32/2 | 0.094 |
|  | 16/0.25 | 32/4 | 0.141 |
| Nalidixic Acid | >128/8 | 32/2 | 0.094 |
|  | >128/4 | 32/4 | 0.141 |
| Neomycin | 2/2 | 32/2 | 1.063 |
|  | 2/4 | 32/4 | 2.125 |
| Nisin | 128/8 | 32/2 | 0.125 |
|  | 128/4 | 32/4 | 0.156 |
| Novobiocin | >128/0.5 | 32/2 | 0.064 |
|  | >128/0.5 | 32/4 | 0.064 |
| Ofloxacin | 1/0.125 | 32/2 | 0.188 |
|  | 1/0.03125 | 32/4 | 0.156 |
| Oxacillin | >128/>128 | 32/2 | 1.063 |
|  | >128/>128 | 32/4 | 1.125 |
| Pentamidine | 128/128 | 32/2 | 1.063 |
|  | 128/128 | 32/4 | 1.125 |
| Polymyxin B | 0.25/0.004 | 32/2 | 0.079 |
|  | 0.25/0.001 | 32/4 | 0.129 |
| Rifabutin | 32/0.0625 | 32/2 | 0.064 |
|  | 32/0.03125 | 32/4 | 0.126 |
| Rifampicin | 32/0.0625 | 32/2 | 0.064 |
|  | 32/0.015625 | 32/4 | 0.125 |
| Rifapentine | 32/0.125 | 32/2 | 0.066 |
|  | 32/0.03125 | 32/4 | 0.126 |
| Rifaximin | 16/0.0625 | 32/2 | 0.066 |
|  | 16/0.03125 | 32/4 | 0.127 |
| Streptomycin | 64/64 | 32/2 | 1.063 |
|  | 64/64 | 32/4 | 1.125 |
| Tetracycline | 32/4 | 32/2 | 0.188 |
|  | 32/4 | 32/4 | 0.250 |
| Tigecycline | 4/1 | 32/2 | 0.313 |
|  | 4/1 | 32/4 | 0.375 |
| Trimethoprim | >128/32 | 32/2 | 0.188 |
|  | >128/16 | 32/4 | 0.188 |

MICa: the MIC of antibiotics, MICb: the MIC of D-11, MICac: the MIC for the antibiotic in the combination, MICbc: the MIC for D-11 in the combination, the synergy effects were bolded in FICI. Red marks mean synergistic effects.
