# Supplementary material for: Elucidating the mechanism by which synthetic helper peptides sensitize Pseudomonas aeruginosa to multiple antibiotics
Source: PLoS Pathog. 2021 Sep 3;17(9):e1009909. doi: 10.1371/journal.ppat.1009909 (PMC8445441; doi:10.1371/journal.ppat.1009909)
Supplement: S5 Table — (DOCX) [file ppat.1009909.s012.docx]

**S5 Table. Bacterial strains, plasmids and primers used in this study.**

| **Strain/ Plasmid /Primer** | | **Description** | **Source (Reference)** |
| --- | --- | --- | --- |
| ***E. coli*** |  | |  |
| DH5α | *F^-^ endA1 glnV44 thi-1 recA1 relA1 gyrA96 deoR nupG purB20* φ80d*lacZ*ΔM15 Δ(*lacZYA-argF*)U169, *hsdR17* (r _K_^–^m_K_^+^), λ^-^ | | Thermo Fisher Scientific |
| ***P. aeruginosa*** |  | |  |
| PA14 | Wild type strain of *Pseudomonas aeruginosa* | | [1] |
| PAO1 | Wild type strain of *Pseudomonas aeruginosa* | | [2] |
| PAK | Wild type strain of *Pseudomonas aeruginosa* | | [3] |
| ATCC27853 | Wild type strain of *Pseudomonas aeruginosa* | | ATCC |
| NR-31040 | Clinical isolate | | Bei resources strain |
| NR-31041 | Clinical isolate | | Bei resources strain |
| HM-214 | Clinical isolate | | Bei resources strain |
| AUMC-Pa-1 | Clinical isolate | | AUMC |
| AUMC-Pa-2 | Clinical isolate | | AUMC |
| AUMC-Pa-3 | Clinical isolate | | AUMC |
| AUMC-Pa-4 | Clinical isolate | | AUMC |
| AUMC-Pa-5 | Clinical isolate | | AUMC |
| AUMC-Pa-6 | Clinical isolate | | AUMC |
| **Plasmids** |  | |  |
| pUCP20 | *Escherichia–Pseudomonas* shuttle vector with *lac* promoter; AMP | | [4] |
| pUCP20-*lacZ* | Expression of *lacZ* by *lac* promoter on pUCP20; CAR | | This study |
| **Primer** | **Sequence (5’→3’)** | | **Function** |
| USER-pUCP20-F | AGCTCGAATUCGTAATCATGGTCATAGCT | | *lacZ* expression |
| USER-pUCP20-R | ATCCTCTAGAGUCGTCCTGCAGG | | *lacZ* expression |
| USER-LacZ-FW | AATTCGAGCUCCACTTGGCTATAATCCGGCG | | *lacZ* expression |
| USER-LacZ-RV | ACTCTAGAGGAUTTATTTTTGACACCAGACCAACTGGTAATGG | | *lacZ* expression |
| lacZ-RT-F | AGGGTGAGTGACGCTGGCA | | RT-PCR |
| lacZ-RT-R | ACTCCAACGCAGCACCAT | | RT-PCR |
| RpsL-RT-F | GTAAGGTATGCCGTGTACG | | RT-PCR |
| RpsL-RT-R | CACTACGCTGTGCTCTTG | | RT-PCR |

The clinical isolate *P. aeruginosa* used were obtained through BEI Resources (NIAID, NIH) and Amsterdam University Medical Center (AUMC), AMP, ampicillin resistance; CAR, carbenicillin resistance.

1. Liberati NT, Urbach JM, Miyata S, Lee DG, Drenkard E, Wu G, et al. An ordered, nonredundant library of *Pseudomonas aeruginosa* strain PA14 transposon insertion mutants. Proc Natl Acad Sci U S A. 2006;103(8):2833-8. Epub 2006/02/16. doi: 10.1073/pnas.0511100103. PubMed PMID: 16477005; PubMed Central PMCID: PMCPMC1413827.

2. Holloway BW. Genetic recombination in *Pseudomonas aeruginosa*. J Gen Microbiol. 1955;13(3):572-81. Epub 1955/12/01. doi: 10.1099/00221287-13-3-572. PubMed PMID: 13278508.

3. Takeya K, Amako K. A rod-shaped Pseudomonas phage. Virology. 1966;28(1):163-5. Epub 1966/01/01. doi: 10.1016/0042-6822(66)90317-5. PubMed PMID: 4955194.

4. Choi KH, Schweizer HP. mini-Tn7 insertion in bacteria with single attTn7 sites: example *Pseudomonas aeruginosa*. Nat Protoc. 2006;1(1):153-61. Epub 2007/04/05. doi: 10.1038/nprot.2006.24. PubMed PMID: 17406227.
