# Supplementary material for: Elucidating the mechanism by which synthetic helper peptides sensitize Pseudomonas aeruginosa to multiple antibiotics
Source: PLoS Pathog. 2021 Sep 3;17(9):e1009909. doi: 10.1371/journal.ppat.1009909 (PMC8445441; doi:10.1371/journal.ppat.1009909)
Supplement: S6 Table — (DOCX) [file ppat.1009909.s013.docx]

**S6 Table. The antibiotic sensibility of the clinical *P. aeruginosa* isolates.**

| Antibiotics (μM) | NR-31040 | NR-31041 | HM-214 | AUMC-Pa-1 | AUMC-Pa-2 | AUMC-Pa-3 | AUMC-Pa-4 | AUMC-Pa-5 | AUMC-Pa-6 |
| --- | --- | --- | --- | --- | --- | --- | --- | --- | --- |
| Amikacin | 4 | 2 | 1 | 1 | 0.25 | 0.0625 | 1 | 1 | 2 |
| Amoxicillin | >128 | >128 | >128 | >128 | >128 | >128 | >128 | >128 | >128 |
| Ampicillin | >128 | >128 | >128 | >128 | >128 | >128 | >128 | >128 | >128 |
| Azithromycin | 128 | 128 | 128 | 128 | 128 | 128 | 128 | 128 | 128 |
| Aztreonam | 4 | 64 | 16 | 32 | 4 | 16 | 32 | 16 | 16 |
| Bacitracin | >128 | >128 | >128 | >128 | >128 | >128 | >128 | >128 | >128 |
| Carbenicillin | 64 | 128 | 128 | >128 | 64 | 128 | 128 | 128 | 128 |
| Cefepime | 4 | 16 | 128 | 8 | 0.5 | 2 | 2 | 4 | 4 |
| Cephalexin | >128 | >128 | >128 | >128 | >128 | 128 | >128 | >128 | >128 |
| Chloramphenicol | 128 | 128 | 128 | >128 | 128 | 128 | 128 | 128 | 128 |
| Chlorobiocin | 1 | 1 | 1 | 4 | 0.25 | 0.5 | 0.25 | 1 | 0.25 |
| Ciprofloxacin | 0.25 | 0.5 | 0.5 | 4 | 0.125 | 0.25 | 0.125 | 0.25 | 0.125 |
| Clarithromycin | 128 | 128 | 128 | 128 | 128 | 128 | 128 | >128 | 128 |
| Colistin | 1 | 0.125 | 1 | 0.5 | 0.5 | 0.25 | 8 | 4 | 4 |
| Coumermycin A1 | 8 | 8 | 8 | 8 | 8 | 8 | 8 | 8 | 8 |
| Doxycycline | 16 | 16 | 16 | 32 | 8 | 16 | 16 | 8 | 16 |
| Eravacycline | 8 | 8 | 8 | 16 | 8 | 8 | 16 | 8 | 8 |
| Erythromycin | 128 | 128 | 128 | 128 | 128 | 128 | 128 | 128 | 128 |
| Fosfomycin | >128 | >128 | >128 | >128 | 128 | >128 | >128 | >128 | >128 |
| Fusaric Acid | >128 | >128 | >128 | >128 | >128 | >128 | >128 | >128 | >128 |
| Gentamicin | 32 | 32 | 1 | 0.5 | 0.25 | 0.0625 | 0.5 | 0.5 | 2 |
| Kanamycin | 128 | 128 | 128 | 64 | 16 | 32 | 32 | 32 | 32 |
| Levofloxacin | 1 | 1 | 1 | 8 | 0.5 | 0.5 | 0.5 | 0.5 | 0.25 |
| Linezolid | >128 | >128 | >128 | >128 | >128 | >128 | >128 | >128 | >128 |
| Loperamide | >128 | >128 | >128 | >128 | >128 | >128 | >128 | >128 | >128 |
| Meropenem | 2 | 4 | 0.25 | 1 | 1 | 0.25 | 2 | 0.25 | 2 |
| Metronidazole | >128 | >128 | >128 | >128 | >128 | >128 | >128 | >128 | >128 |
| Minocycline | 16 | 16 | 16 | 32 | 8 | 16 | 16 | 16 | 16 |
| Nalidixic Acid | >128 | >128 | >128 | >128 | >128 | >128 | >128 | >128 | >128 |
| Neomycin | 8 | 4 | 2 | 1 | 0.5 | 0.0625 | 0.5 | 0.5 | 2 |
| Nisin | >128 | >128 | >128 | 128 | 128 | 128 | >128 | >128 | >128 |
| Novobiocin | >128 | >128 | >128 | >128 | >128 | >128 | >128 | >128 | >128 |
| Ofloxacin | 2 | 2 | 2 | 32 | 1 | 1 | 1 | 1 | 0.5 |
| Oxacillin | >128 | >128 | >128 | >128 | >128 | >128 | >128 | >128 | >128 |
| Ofloxacin | >128 | >128 | >128 | >128 | >128 | 128 | >128 | >128 | >128 |
| Polymyxin B | 0.25 | 0.125 | 0.25 | 0.25 | 0.25 | 0.125 | 1 | 1 | 1 |
| Rifabutin | 32 | 32 | 32 | 32 | 8 | 16 | 16 | 32 | 16 |
| Rifampicin | 16 | 32 | 16 | 32 | 16 | 16 | 16 | 32 | 32 |
| Rifapentine | 8 | 16 | 8 | 16 | 8 | 8 | 16 | 32 | 16 |
| Rifaximin | 16 | 16 | 8 | 32 | 8 | 8 | 8 | 32 | 16 |
| Spiramycin | >128 | >128 | >128 | >128 | >128 | >128 | >128 | >128 | >128 |
| Streptomycin | >128 | >128 | 128 | 16 | 8 | 128 | 8 | 8 | 16 |
| Telithromycin | 128 | 128 | 128 | 128 | 64 | 64 | 64 | 64 | 64 |
| Tetracycline | 64 | 64 | 64 | 128 | 64 | 64 | 128 | 64 | 64 |
| Tigecycline | 8 | 8 | 4 | 8 | 2 | 4 | 8 | 8 | 8 |
| Trimethoprim | >128 | >128 | >128 | >128 | >128 | >128 | >128 | >128 | >128 |
