# Supplementary material for: Elucidating the mechanism by which synthetic helper peptides sensitize Pseudomonas aeruginosa to multiple antibiotics
Source: PLoS Pathog. 2021 Sep 3;17(9):e1009909. doi: 10.1371/journal.ppat.1009909 (PMC8445441; doi:10.1371/journal.ppat.1009909)
Supplement: Attachment — Submitted filename: Reviewer plos pathgen.docx [file ppat.1009909.s014.docx]

Reviewer’s comments

This paper examines the antibacterial mechanism of a synthetic antimicrobial peptide, D-11, against P. aeruginosa and its synergistic effect with azithromycin. This is the quality research, but I have some minor concerns as follows.

- Non-natural modifications such as d-amino acid substitutions are widely introduced in peptides to enhance their therapeutic efficacy however there were concerns or issues on the clinical immunogenicity of the d-amino acid peptide. The author should provide data or information to support the clinical use as new therapeutics.
- Several peptides (previously reported or newly designed peptides) were selected for combination study with azithromycin. Is there any reason or criteria to choose these candidate peptides and their modifications? Provide a defined reason would help the readers to understand more.
- Why the concentration of azithromycin used in biofilm formation inhibition and killing efficiency on established biofilms were not equal while the concentration of D-11 was fixed at the same dose?
- The ability to inhibit the biofilm formation of peptide might be from the killing activity or via intracellular mechanism such as down regulation of biofilm gene expression. As the concentration used is lower than MIC therefore the major effect is involved with the down regulation of the biofilm associated gene expression. The author should provide more explanations and correlate the result with transcriptome analysis data.
- D-11 showed strong synergistic effects with 25 antibiotics, why only 4 antibiotics were selected for ex vivo bacteremia model and 3 antibiotics for in vivo. The FICI of other antibiotics are lower than that of four drugs.
- In vivo experiment showed that the combinations of D-11 with azithromycin, chloramphenicol and doxycycline could reduce more than 2-3 log10 CFU in the abscess compared to each individual antibiotic. The mechanism of action of three drugs (azithromycin, chloramphenicol and doxycycline) are involved with inhibition of protein synthesis (translation). The author should provide more discussions on in vivo and ex vivo results based on mechanism of action of antibiotics.
- More data on the synergistic effect of D-11 with antibiotics against clinically isolates especially MDR and XDR P. aeruginosa would strongly support the use of D-11 as an antibiotic adjuvant.
