# Supplementary material for: Elucidating the mechanism by which synthetic helper peptides sensitize Pseudomonas aeruginosa to multiple antibiotics
Source: PLoS Pathog. 2021 Sep 3;17(9):e1009909. doi: 10.1371/journal.ppat.1009909 (PMC8445441; doi:10.1371/journal.ppat.1009909)
Supplement: Attachment — Submitted filename: Response to reviewers.docx [file ppat.1009909.s015.docx]

Dear Editor:

We thank both you and the reviewers for the attention given to our manuscript entitled “Elucidating the mechanism by which synthetic helper peptides sensitize *Pseudomonas aeruginosa* to multiple antibiotics” (PPATHOGENS-D-21-00712). The constructive comments of the reviewers are valuable and very well taken. The following is a point-by-point synopsis of the changes we have made to address the comments and concerns raised by the reviewers.

Point by point response to the reviews:

**Part II – Major Issues: Key Experiments Required for Acceptance**

Please use this section to detail the key new experiments or modifications of existing experiments that should be absolutely required to validate study conclusions.

Generally, there should be no more than 3 such required experiments or major modifications for a "Major Revision" recommendation. If more than 3 experiments are necessary to validate the study conclusions, then you are encouraged to recommend "Reject".

**Reviewer #1**

**1) Synergy is based on classic broth dilution assays (including checkerboard) which have a very non-detailed readout after 18h incubation. Most antimicrobial peptides work within 1-2 hours in such assays and then lose activity (from most kinetic figures in this manuscript the effect of D-11 also seems to be mostly within the first hour). Therefore, a sub-MIC concentration can (and will) kill a substantial amount of bacteria but the residual number of bacteria (in theory 1 viable bacterium is enough) will grow out to a dense population after 18h. The authors should therefore determine what actually happens in the first hours with respect to bactericidal activity.**

Response: According to the reviewer suggestion, we have determined the killing kinetics for the peptide D-11 at 0, 2, 4, 8, 16, 32 μM in 6 hours using MHB medium with 10^6^ CFU/mL suspension cells (as recommended for the MIC test by the CLSI). The results showed that the sub-MIC concentration of D-11 cannot kill the bacteria and only with the higher (32 μM) concentration could D-11 kill the bacteria. When a sub-MIC concentration of D-11 (4 μM) was combined with AZM, the combination treatment can kill the bacteria efficiently, while the mono-treatment cannot kill the bacteria. We added this part of results and discussion in the manuscript (Fig. 1).

**Fig. 1** (A) Time killing curves for D-11 against PAO1 during 6 h incubation at the indicated concentration. (B) Time killing curves for D-11 and AZM monotherapy and combination therapy against PAO1 during 24 h incubation at the indicated concentration. Error bars represent mean ± SD for three biological replicates.

**2) All effects described for D-11 alone, are very similar to many native antimicrobial peptides, including LL-37. This or similar cathelicidins should have been taken along as control. Now I don’t see any indication why D-11 is different from LL-37 (or many other cathelicidins).**

Response: As stated in the manuscript, D-11 is a close-to-nature fragment of the human cathelicidin LL-37. It is a shorter peptide, containing only D-amino acids, with also a couple of differences to the native sequence of LL-37, which provides a more cationic charge that may enhance the cell penetrating activity:

LL-37	LLGDFFRKSKEKIGKEFKRIVQRIKDFLRNLVPRTES-OH

D-11	------------------RIVQRIK**KW**LR---------NH_2_

In the manuscript, we have tested other 5 variants of this peptide (KR-12-a2, L-11, D-11R, L-11-R5W, D-11-k(r)), getting a similar synergistic effect (except for L-11-R5W which was more active individually but it failed in the synergism). Besides, D-11 is an optimization of the peptide KR-12-a2 selected among other variants to increase the synergistic activity of lipid II binding antibiotics (Li et al., 2021: 10.1038/s42003-020-01511-1.). According to the suggestions of the reviewer we tested the full length cathelicidin peptide LL-37 activity and the synergistic effect in combination with azithromycin. LL-37 is more active individually (MIC=8μM) but it does not show a synergistic effect with azithromycin. We included this part of results in the manuscript (Table 1).

**3) Transcriptome experiments are done with 8uM Azithromycin. This should have been done at 4 μM since this is the presumed concentration where synergy occurred.**

Response: We did this because the MIC test was performed under the condition of 10^6 cells. Considering the higher number of cells (~5*10^8) used in the transcriptome experiment and the relatively short time of stress exposure, we used 8 μM to be sure to get enough differences in genes expression. In our opinion, it is not to be expected to see big differences in transcriptomes using either 4 or 8µM and neither an unexpected effect on the rest of the assays performed. To prove it, we have checked the survival rate of the cells after the treatment, and there are not significant differences between 4μM and 8μM AZM combined with D-11 (Figure 2A). Besides we have checked the gene expression of selected genes based on the transcriptomic results under different treatments (4 or 8 µM of AZM with and without D-11) by RT-PCR. The results showed that there is no difference between 4μM and 8μM AZM combined with D-11 (Figure 2B).

**Fig. 2** (A) Time killing curves for D-11 against PAO1 during 6 h incubation at the indicated concentration. (B) Time killing curves for D-11 and AZM monotherapy and combination therapy against PAO1 during 24 h incubation at the indicated concentration. Error bars represent mean ± SD for three biological replicates.

**4) Biofilm experiments are done very simplistic and lacks characterization. Specifically: a) The authors should show and confirm that a proper biofilm is formed (not just 1 or 2 layers of adhered bacteria to glass). b) Biofilm growth has several stages; it is custom to have an adherence phase of 8 h then replace the supernatant with new media and let the initial biofilm grow out. c) Crystal violet detection of biofilm mass should be included to get a second readout: biofilm mass (including dead bacteria) and number of viable bacteria in the biofilm. d) When treatment of a preformed biofilm results in lower number of viable bacteria, are these bacteria killed or just released in the supernatant because the biofilm is broken off. The authors should check this because the latter would actually be a bad characteristic that could increase spreading of biofilms in an in vivo setting.**

Response: We have now grown the biofilm following the reviewer’s suggestion and confirmed the formation of biofilm by a crystal violet stain (Fig. S5). We also included the results of crystal violet detection in the manuscript (Fig. 5A). For the treatment of the preformed biofilm, we reperformed the experiment and determined the live bacteria number in the supernatant. We included the results, which were in accordance with the previous ones, in the manuscript (Fig. 5D).

**Reviewer #2: (No Response)**

**Reviewer #3: There was no major issue.**

**Part III – Minor Issues: Editorial and Data Presentation Modifications**
Please use this section for editorial suggestions as well as relatively minor modifications of existing data that would enhance clarity.

**Reviewer #1:**

**Minor:**
**1) Please provide purity of peptides.**

Response: All of the peptides had a purity higher than 95%. The description was added to the Materials and Methods section.

**2) Fig4C: since this is not an antibiofilm graph I would give this a different spot in the article.**

Response: We reorganized the positions of the figures.

**3) Line 180-193: It is not clear (and unlikely) that the interaction with bacterial components is very specific except for the pure electrostatic interaction. If the authors would add another (nonbacterial) negatively charged compound a similar effect would be seen I predict. Such controls should be added to the manuscript.**

Response: The synergistic effect of D-11 is based on its activity on an intracellular target that induces ROS accumulation. To reach this target, the peptide has to cross the membrane (as a cell penetrating peptide) and thus, interaction with the lipids is required. The goal of this point was to confirm that the peptide was able to interact with the main phospholipids of the outer and inner membranes of *P. aeruginosa* and the absence of interaction with eukaryotic cell membrane components. Electrostatic interaction is expectable due to charge of the peptide and necessary for the activity of the peptide. We used the nonbacterial negatively charged compound BSA to test the MIC of D-11. In the presence of BSA, the MIC of D-11 is not changed even at a high concentration (128 mg/L) (Fig. 2D). To this must be added that the synergistic effect was neither impaired in a complex matrix as blood, which is quite rich in negatively charged proteins.

**4) Line 170 (and more spots in this paragraph): the figures seem to have been renumbered. Here it says Fig 1D instead of 3D**

Response: We corrected these mistakes.

**5) Fig1H in ploymixin should read polymyxin**

Response: We corrected the mistake.

**6) Fig 1H The effect of the control polymyxin seems relatively mild. Another control leading to 100% loss of membrane potential should be included.**

Response: Following the suggestion, we used SDS as a positive control to reach the max depolarization and updated the results (Fig. 2H).

**7) L592: Bacterial should read bacteria.**

Response: We corrected this mistake.

**8) Line 149: I thought ketolides are a subgroup of macrolides? And why would it be expected that ketolides are not synergistically acting? Or am I misreading this sentence?**

Response: We agree that the ketolides belong to the macrolides. For spiramycin, the peptides do not show the synergistic effects. We think this may be due to the fact that the activity of this antibiotic itself is too weak against *P. aeruginosa.* The MIC of spiramycin alone is higher than 128 μM, and the MIC of spiramycin reached 128 μM in the presence of D-11. When we used 256 for the calculation of FICI, the value did not reach synergy (FICI=0.625). (For example, if the MIC of spiramycin would be 1024, the FICI = 128/1024 + 4/32 = 0.25, and it should be synergy). We revised the sentence.

**9)Line 168: How should PI uptake be interpreted: inner membrane permeabilization (which can synergize with macrolide action), or just killing the bacterium??? In which case it has nothing to do synergy.**

Response: We checked the survival rate of the bacteria under the same condition; the results showed that the sub-MIC of D-11 cannot kill the bacteria, and only the 32 μM D-11 and the PB treatment is resulting in death of the cells. The results indicated that the increase of PI uptake was mainly due to the inner membrane permeabilization at the sub-MIC D-11 treatment. We added this result to the supplementary Fig. S3 and discuss it in the manuscript.

**10) Fig 4A: Biofilm formation: what is exactly expressed on the y-axis, CFU/OD. What does that mean exactly?**

Response: We apology for the misunderstood. Since the bacteria growth rate is different under different treatment conditions, we normalized the data dividing the CFU/ml between OD_600_. Previous studies have shown that bacterial growth rates should be considered when quantifying biofilms (Naves et al., 2008, 10.1111/j.1365-2672.2008.03791.x; Niu and Gilbert, 2004, 10.1128/AEM.70.12.6951-6956.2004;). So, we divided the final density of planktonic bacteria to indicate the relative biofilm formation. Now, we did the experiments following the reviewer’s suggestion and included the new results to the manuscript.

**11) Fig 4B If 16 uM Azithromycin already reduces the biofilm by 99% (2LOG) what does a further reduction by D-11 really mean? How relevant is this? Please explain**

Response: It is well known that a sub-MIC concentration of azithromycin can retard *P. aeruginosa* biofilm formation by interfering with cell-to-cell signaling (Richard J Gillis. 2004, 10.1128/JCM.42.12.5842-5845. Kenji Nagino, 10.1111/j.1469-0691.1997.tb00279.). In our study, D-11 can increase the activity of azithromycin against *P. aeruginosa*. In this result, the level of biofilm reduction is calculated based on the total number of viable bacteria attached to the surface. Azithromycin can inhibit the biofilm formation no matter by either killing attached bacteria or inhibiting the synthesis of bacterial biofilm forming components. From our results, the bactericidal effect of the combination is significantly better than that of the single treatment. And our transcriptomic results indicate that the expression of biofilm formation related genes in the co-treatment group were significantly downregulated compared with the azithromycin treatment alone. On the one hand, using the peptide as adjuvant can enhance the antibacterial ability of azithromycin, and on the other hand, it can reduce the dose of azithromycin needed and help slow down the development of drug resistance.

**12) Fig 4D: Activity in blood: it is a nice visual, but why aren’t actual CFU/ml calculated and provided? From the images it seems as if D-11 at 4uM already results in substantial partial killing of *P. aeruginosa* (compare top line of 0 and 4uM, last three dilutions.**

Response: Because we cannot count the exact cell number under the treatment of some combinations with a high concentration of antibiotic, since this killed the bacteria too much. And with the antibiotic treatment alone or in the non-treated group, bacteria grew too much. So, we now prepared the results as Fig. 4D (Now Fig.7A ) and S6 to show the strong synergistic effects in blood.

Compare the D-11 treatment alone after 4 h (The top line of 4μM) with the initial cell number at 0 h (the first line of the figure). The result indicates that the sub-MIC D-11 treatment cannot control the infection (the bacteria grow). As the reviewer pointed out, compared to the top line of 0 and 4 μM, this just indicated the D-11 treatment can slow down the growth of bacteria in the blood compare with no treatment. But the bacteria still grow a bit compared with the first line (initial bacteria number). To avoid any misunderstanding, we changed the time label and used red color and bolded it.

**13) the activity in blood clearly shows that sub-MIC D-11 has a clear bactericidal effect . Is the synergy that is measured therefore not just an effect of a lowered number of viable bacteria that have to be killed by the second component (azithromycin)? The MIC of azithromycin at much lower bacterial densities should be determined.**

Response: As explained above, the sub-MIC D-11 does not show a bactericidal effect and we also confirmed the synergy effect is not because of the killing of individual components (Fig.1B). In addition, we also tested the MIC of azithromycin at lower bacterial densities.

| Initial bacterial densities | 10^6 CFU/mL | 10^5 CFU/mL | 10^4 CFU/mL | 10^3 CFU/mL |
| --- | --- | --- | --- | --- |
| MIC of AZM (μM) | 128 | 128 | 64 | 64 |

The result indicates that even at the lower bacterial densities, the MIC of AZM only reduced two times. The MIC for AZM was reduced 32 times when combined with 4 µM D-11, indicating a true synergistic effect.

**14) Different experiments are carried out with very different bacterial densities, ranging from 1x10^6 CFU/ml (MIC assay) to an OD=0.5 corresponding to >10^8 CFU/ml. I understand that higher densities are needed to detect fluorescent signals for membrane potential for example, but the antibacterial effect will be different at these largely different densities and one cannot compare and relate the effect of 4 uM D-11 in different assays. Please clarify this lack of consistency and its potential effect on interpretation in the manuscript.**

Response: For the MIC assays, the bacterial density used for the MIC test is the one recommended by CLSI for such experiments. As indicated by the reviewer, in other cases higher CFU/ml were considered, just following established protocols and to provide measurable results. Of course, the antibacterial effect of D-11 could be different if more cells are present but the action mechanism (that we are determining) should be the same in any case. Besides, in such experiments, several concentrations of D-11 are tested providing dose-response results that improve understanding of the results. Also, we would like to indicate that the observed results are connected and explained and that several methodologies were performed to demonstrate them.

**15) The interpretation of the checkerboard assay is unclear to me. Is synergy observed over the whole concentration spectrum of both compounds or (taking D-11 as an example) only at this very specific combination of 4uM Azithromycin and 4uM of D-11?
The FIC is 0.156 based on these partial MICs (4/128 + 4/32), but for example…... how much azithromycin was needed at a D-11 concentration of 2 uM and was that combination synergistic as well. (And that goes for all tested concentrations of course). Please explain.**

Response: For the initial screening of a synergistic effect, we first tested the MIC of the peptides alone, and next, 8 μM antimicrobial peptide was used in combination with azithromycin, while the MIC of the peptide is higher than 32 μM. If not, the 1/4 MIC of the peptide was used in the combination. If the peptide showed a synergistic effect with azithromycin, a standard checkerboard broth microdilution test was performed to test the synergy of the combined antimicrobial agent. Finally, we selected the relative lower FICI combinations shown in Table 1. In the Table S1, we listed the synergy results with lower concentrations of peptides combined with all the macrolides and the synergy effects were indicated in Fig. S1. We now describe it more clearly in the Method section.

**Reviewer #2:** The following minor issues should be addressed to improve the manuscript:

**1) Introduction: Include a description of the peptides that are described in the study (Table 1), and more specially provide any state of the art related to similar studies. Background information should be provided in the introduction regarding these peptides to put the study in context. It is not mentioned where the EC5, KR-12 and L-11 and the rest of peptides in table 1 originate from, references are only provided in the table.**

Response: We added this part of introduction to the manuscript.

**2) Table 1: it is confusing to read and can be misleading regarding some of the data. Change order of the columns, including first the MIC of the peptides alone and in combination with azithromycin, then the MIC of azithromycin and in combination with the peptide.**

Response: We changed this table to make it more clear.

**3) Line 121: Provide clarification regarding the meaning of “reverse sequence of the amino acid”**

Response: We have corrected this sentence to make it clearer.

**4) Line 151: Table 2 is not included in the main text**

Response: We renumbered the tables and ensured that they are in the correct positions.

**5) Line 260: include the concentration used for the combination of D-11 and azithromycin in the transcriptome study. It is mentioned in line 271, but should be included when describing the experiment**

Response: We included the concentration of D-11 and azithromycin at the beginning.

**6) Line 264: tables 4-6 are not provided in main text**

Response: We correct this mistake and put the tables in the supplementary results (S2-4 Table). Because the tables are too big, we put them in an excel file.

**7) Line 325 Tables 3 is not provided in main text**

Response: We correct this mistake and put it in the supplementary results (S5 Table).

**8) Efficacy in murine model: no information is provided regarding the concentration and dosage of the combination with different antibiotics in the text. Fig 4 E-G: include the concentration units in all the graphics**

Response: We included this information in the main text and the figure legend.

**Reviewer #3:**

Reviewer’s comments

This paper examines the antibacterial mechanism of a synthetic antimicrobial peptide, D-11, against *P. aeruginosa* and its synergistic effect with azithromycin. This is the quality research, but I have some minor concerns as follows.

**1) Non-natural modifications such as d-amino acid substitutions are widely introduced in peptides to enhance their therapeutic efficacy however there were concerns or issues on the clinical immunogenicity of the d-amino acid peptide. The author should provide data or information to support the clinical use as new therapeutics.**

Response: This comment is valuable. In our previous study, we demonstrated that D-11 is not toxic to HEK-293 cells and has no hemolytic activity even at high concentrations (Li et al., 2021: 10.1038/s42003-020-01511-1.). We believe that it is a safe drug candidate. No obvious side effects were observed at the effective concentration of 10 mg/kg in the *in vivo* test. However, we still carried out high-dose toxicity tests in mice according to the recommendations. We found that subcutaneous injection of 20 mg/kg of D-11 was slightly irritating, while high-dose (50 mg/kg) D-11 has strong irritation and can cause skin tissue damage post injection 24 hours in mice. Although 50 mg/kg is much higher than the effective concentration (10 mg/kg), it`s still a concern for practical applications. Therefore, we will continue to conduct related research in the future. We also discussed this concern in the discussion section.

**Fig. 3.** Appearance of mice after 24 hours of subcutaneous injection indicated concentration of D-11.

**2) Several peptides (previously reported or newly designed peptides) were selected for combination study with azithromycin. Is there any reason or criteria to choose these candidate peptides and their modifications? Provide a defined reason would help the readers to understand more.**

Response: We selected these peptides based on their activities against Gram-negative pathogens, the low human cell toxicity and their variants were modified based on increasing the stability, amphiphilicity and hydrophobicity. We added this description in the main text.

**3) Why the concentration of azithromycin used in biofilm formation inhibition and killing efficiency on established biofilms were not equal while the concentration of D-11 was fixed at the same dose?**

Response: When tested the inhibition of biofilm formation a relative low concentration of azithromycin was used, because we didn`t want the drug to kill too much cells. For the biofilm killing assay, we selected a relative higher concentration to kill the bacteria inside of the biofilm, because the preformed biofilm is highly resistance to the antibiotics. For the concentration of D-11, 2-4 μM is enough to show a synergistic effect with azithromycin, so we continued to use this concentration.

**4) The ability to inhibit the biofilm formation of peptide might be from the killing activity or via intracellular mechanism such as down regulation of biofilm gene expression. As the concentration used is lower than MIC therefore the major effect is involved with the down regulation of the biofilm associated gene expression. The author should provide more explanations and correlate the result with transcriptome analysis data.**

Response: We discussed this in the main text.

**5) D-11 showed strong synergistic effects with 25 antibiotics, why only 4 antibiotics were selected for ex vivo bacteremia model and 3 antibiotics for in vivo. The FICI of other antibiotics are lower than that of four drugs.**

Response: We tested all the active macrolides and some other groups of antibiotics combined with D-11 in the *ex vivo* bacteremia model. Some of the results are in the supplementary (Fig. S6 and S7). The selections for *in vivo* test were based on the *ex vivo* results. As shown in Fig. S7, a single dose of azithromycin, clarithromycin, chloramphenicol and doxycycline can control the blood infection more than 24 hours when combined with D-11. Both azithromycin and clarithromycin belong to the macrolides. So, we selected azithromycin, chloramphenicol and doxycycline for the *in vivo* tests.

**6) In vivo experiment showed that the combinations of D-11 with azithromycin, chloramphenicol and doxycycline could reduce more than 2-3 log10 CFU in the abscess compared to each individual antibiotic. The mechanism of action of three drugs (azithromycin, chloramphenicol and doxycycline) are involved with inhibition of protein synthesis (translation). The author should provide more discussions on in vivo and ex vivo results based on mechanism of action of antibiotics.**

Response: We added discussion in the main text.

**7) More data on the synergistic effect of D-11 with antibiotics against clinically isolates especially MDR and XDR P. aeruginosa would strongly support the use of D-11 as an antibiotic adjuvant.**

Response: We collected other six clinical *P. aeruginosa* isolates and tested the synergistic effects of antibiotics and D-11. We added these results in the manuscript. The antibiotic sensibility of these strains was listed in Table S8.
